# Complete chloroplast genome sequence of *Tamarix taklamakanensis* (Tamaricaceae)

**DOI:** 10.1080/23802359.2021.1990807

**Published:** 2021-10-27

**Authors:** Xin-An Pang, Pei-Pei Jiao, Tian-Ge Yang, Hong Liu

**Affiliations:** a Key Laboratory of Protection and Utilization of Biological Resources in Tarim Basin Xinjiang Production and Construction Corps, Tarim University, Alar, China; b College of Life Science, Tarim University, Alar, China; c College of Life Science and Technology of Huazhong Agricultural University, Wuhan, China; d Hubei Provincial Key Laboratory for Protection and Application of Special Plant Germplasm in Wuling Area of China, College of Life Sciences, South-Central University for Nationalities, Wuhan, China

**Keywords:** *Tamarix taklamakanensis*, chloroplast genome, evolution

## Abstract

*Tamarix taklamakanensis* M. T. Liu, belonging to the genus *Tamarix* (family Tamaricaceae), is an endangered shrub endemic to arid basins in northwestern China. Most of species in this genus have high medicinal value. The complete chloroplast genome was reported in this study. The chloroplast genome with a total size of 156,177 bp consists of two inverted repeats (IR, 26,571 bp) separated by a large single-copy region (LSC, 84,778 bp) and a small single-copy region (SSC, 18,257 bp). Further annotation revealed the chloroplast genome contains 106 genes, including 73 protein coding genes, 29 tRNA genes, and 4 rRNA genes. A total of 64 simple sequence repeats (SSRs) were identified in the chloroplast genome. This information will be useful for study on the evolution and genetic diversity of *T. taklamakanensis* in the future.

*Tamarix taklamakanensis* M. T. Liu is an Endangered desert shrub belonging to the genus *Tamarix*, Tamaricaceae, and it often grows in arid desert areas of northwestern China. Since this species shows a strong drought resistant and heat tolerance, it plays an important role in sand fixation and reforestation on mobile sand dunes as a pioneer tree species of Western China (Su et al., 2017, 2018). Study on this species is of scientific significance to the further research on the phylogeny of this genus. In this study, to obtain the new insight into the phylogeny of *T. taklamakanensis*, we sequenced, assembled, and annotated the chloroplast genome.

The materials of *T. taklamakanensis* in this study were collected around the Taklimakan Desert Highway from Aler to Hetian, Xinjiang province of China (80°45.014′E, 38°12.020′N, 1105 m above sea level). The voucher specimen (TD-04001, *Tamarix taklamakanensis* M. T. Liu) was stored in the herbarium of Tarim University. The leaves total genomic DNA was extracted using CTAB method (Doyle and Doyle 1987). Samples yielding at least 1.0 μg DNA was selected for subsequent library construction and de novo sequencing. Genomic DNA of selected samples was used to build paired end libraries with insert sizes of 150 bp. The complete genome of *T. taklamakanensis* was sequenced using the Illumina HiSeq 2500 platform at Frasergen Company (Wuhan, China), yielding approximately 50 Gb of high-quality sequences. First, the clean data was quality-controlled by using FastQC v0.11.9 (http://www.bioinformatics.babraham.ac.uk/projects/fastqc/). Then, the whole chloroplast genome was assembled using GetOrganelle v1.7.3 (Jin et al. 2020). Finally, the final assembly result was obtained, and checked the accuracy of assembly results, the slimmed assembly graph and selected target assembly graph could be visualized by Bandage v0.8.1 (Wick et al. 2015) to assess the completeness of the final graph. Gene annotation was performed using CPGAVAS2 (http://47.96.249.172:16019/analyzer/annotate) (Shi et al. 2019). In addition, we chose *Tamarix chinensis* (MN229512) as the reference genome and used PGA (https://github.com/quxiaojian/PGA) (Qu et al. 2019) for annotation. The different annotations of protein-coding sequences were confirmed using BLASTx in NCBI. The complete chloroplast genome was 156,177 bp (MW125612) and composed of two IRs of 26,571 bp each, which divide a large single copy (LSC) region of 84,778 bp and a small single copy (SSC) region of 18,257 bp, and the average GC content was 36.41%. The chloroplast genome encodes 106 functional genes, including 73 protein-coding genes, 29 tRNA genes, and 4 rRNA genes. A total of 64 SSR markers ranging from mononucleotide to hexa-nucleotide repeat motif were identified in *T. taklamakanensis* chloroplast genome.

In order to explore the phylogenetic relationship of *T. taklamakanensis* within Tamaricaceae, additional 22 species from Caryophyllales were studied. With the *Osyris alba* and *Osyris wightiana* as the outgroups, the phylogenetic tree was built from the joint matrix of whole protein-coding genes by maximum-likelihood (ML) and Bayesian inference (BI) (Figure 1). The ML tree was generated using IQ-TREE v2.1.2 (Nguyen et al. 2015) based on the best model of TVM + F+R4 and 1000 bootstrap replicates, and BI analysis was performed in MrBayes v3.2.7 (Ronquist et al. 2012). This result showed that the analyzed *T. taklamakanensis* was closer to *Tamarix chinensis*. Compared with the genus *Fagopyrum*, the genus *Tamarix* showed closely related to *Myricaria* in the phylogenetic tree.

**Figure 1.**
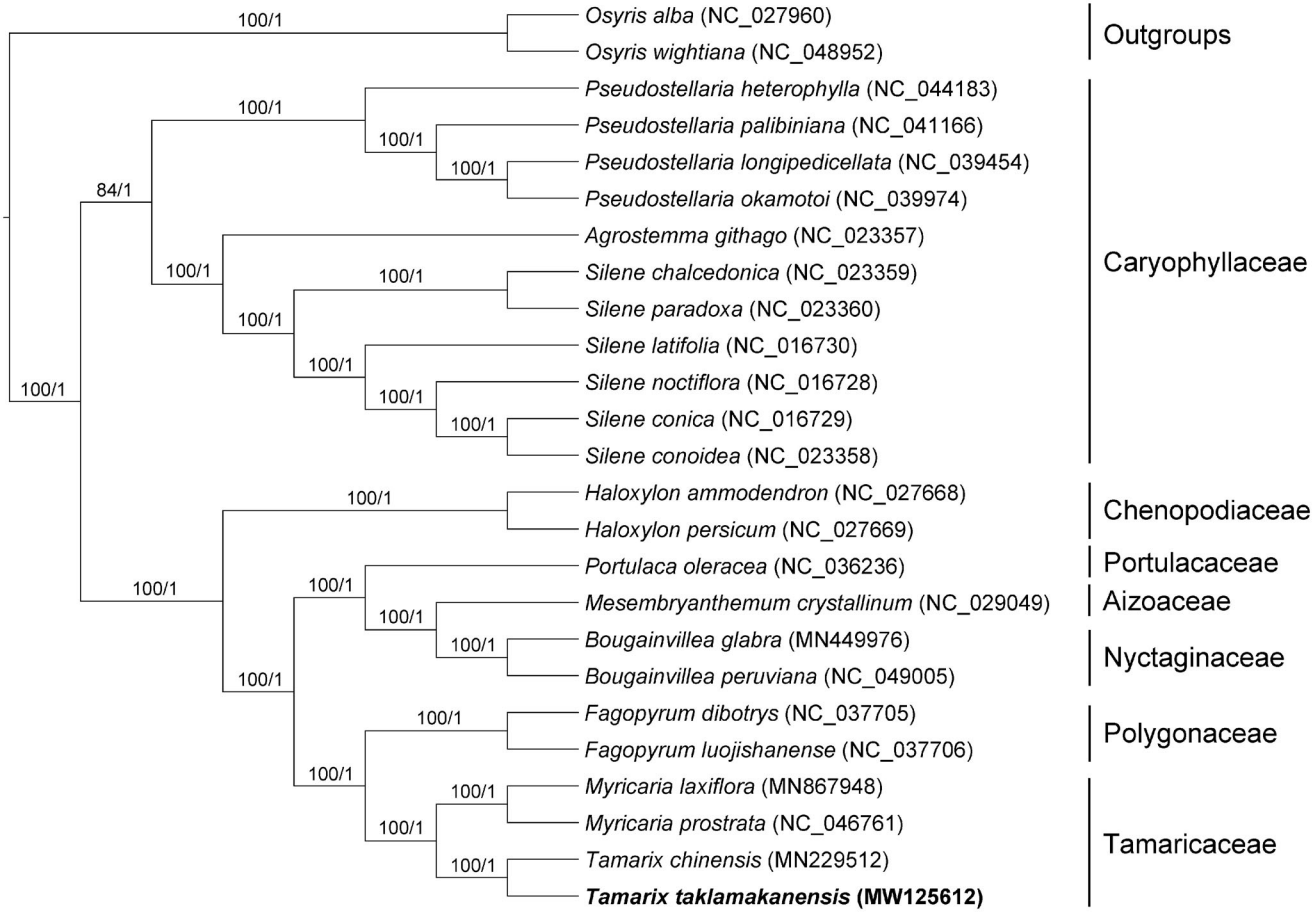
Phylogenetic tree reconstructed by maximum-likelihood (ML) and Bayesian inference (BI) analysis based on the whole chloroplast protein-coding genes of 25 species. Values above branches are maximum likelihood bootstrap percentages (BS) / Bayesian posterior probabilities (PP).

## Data Availability

The genome sequence data that support the findings of this study are openly available in GenBank of NCBI at [https://www.ncbi.nlm.nih.gov] (https://www.ncbi.nlm.nih.gov/) under the accession no. MW125612. The associated “BioProject”, “SRA”, and “Bio-Sample” numbers are PRJNA670259, SRR12881007, and SAMN16491008 respectively.
